# Antipsoric Remedies

**Published:** 1844-07-27

**Authors:** 


ANTIPSORIC REMEDIES.

Various new modes of curing the itch appear from time to time; and if it be true that the thing needful is the destruction of the itch insect, it is probable that some remedy may be brought into general use which may be equally certain, and less noisome, than the common sulphur ointment. M. Dornblueth recommends a combination of two parts of common soap and one of powdered white hellebore, made into a paste with boiling water. This must be daily rubbed into the parts affected, until the itching is succeeded by a burning sensation, after which daily ablutions and clean linen complete the cure.

M. Aubé declares that one friction with oil of turpentine effects a perfect cure; M. Cazenave says the same of a solution of iodine; and it is also stated that all essential oils, especially those of anise and peppermint, possess the same properties.—*Prov. Med. Journ.*, from *Bouchardat's Annuaire de Thérapeutique.*

